# Ethical and legal challenges in nanomedical innovations: a scoping review

**DOI:** 10.3389/fgene.2023.1163392

**Published:** 2023-05-12

**Authors:** Sophia Wasti, Il Ho Lee, Sumin Kim, Jae-Hyun Lee, Hannah Kim

**Affiliations:** ^1^ Asian Institute of Bioethics and Health Law, Yonsei University, Seoul, Republic of Korea; ^2^ Institute for Legal Studies, Yonsei University, Seoul, Republic of Korea; ^3^ Korea National Institute for Bioethics Policy, Seoul, Republic of Korea; ^4^ Advanced Science Institute, Yonsei University, Seoul, Republic of Korea; ^5^ Institute for Basic Science (IBS) Center for Nanomedicine, Seoul, Republic of Korea; ^6^ College of Medicine, Division of Medical Humanities and Social Science, Yonsei University, Seoul, Republic of Korea

**Keywords:** scoping review, nanomedical research, consent to research, patient safety, precautionary principle, regulation of nanomedicines

## Abstract

**Background:** Rapid advancements in research and development related to nanomedical technology raise various ethical and legal challenges in areas relevant to disease detection, diagnosis, and treatment. This study aims to outline the existing literature, covering issues associated with emerging nanomedicine and related clinical research, and identify implications for the responsible advancement and integration of nanomedicine and nanomedical technology throughout medical networks in the future.

**Methods:** A scoping review, designed to cover scientific, ethical, and legal literature associated with nanomedical technology, was conducted, generating and analyzing 27 peer-reviewed articles published between 2007–2020.

**Results:** Results indicate that articles referencing ethical and legal issues related to nanomedical technology were concerned with six key areas: 1) harm exposure and potential risks to health, 2) consent to nano-research, 3) privacy, 4) access to nanomedical technology and potential nanomedical therapies, 5) classification of nanomedical products in relation to the research and development of nanomedical technology, and 6) the precautionary principle as it relates to the research and development of nanomedical technology.

**Conclusion:** This review of the literature suggests that few practical solutions are comprehensive enough to allay the ethical and legal concerns surrounding research and development in fields related to nanomedical technology, especially as it continues to evolve and contribute to future innovations in medicine. It is also clearly apparent that a more coordinated approach is required to ensure global standards of practice governing the study and development of nanomedical technology, especially as discussions surrounding the regulation of nanomedical research throughout the literature are mainly confined to systems of governance in the United States.

## 1 Introduction

Nanotechnology is the design, production, and application of systems, structures, and devices at the nanoscopic scale (Bawa et al., 2005). The development and scope of nanotechnology have witnessed rapid advancements in tandem with other types of digital medical technology, such as machine learning (ML) and artificial intelligence (AI). These developments present material opportunities for the delivery of personalized medicine and healthcare strategies at the nanoscopic scale, especially where advances in ML enable faster, more effective cross-platform communication and better, more accurate interpretations due to the vast amounts of data accumulated by AI systems (Singh et al., 2020a). The nature of these hybrid types of technology has the potential to revolutionize healthcare delivery but presents several legal and ethical challenges. For example, nanomedical devices are increasingly integrated with the so-called “Internet of Things,” such as smartwatches and smartphones, especially in terms of health and medical applications. Additionally, innovations in nanomedical technology related to health monitoring allow doctors to easily monitor glucose levels in blood vessels (El-Din and Manjaiah, 2017) or determine stented vessels in the heart that are blocked by in-stent restenosis via nano-sensors in heart stents (Hoare et al., 2019). Although this review focuses on ethical and legal issues related to nanomedical technology, it recognizes the intimately linked nature of nanomedical technology to AI and ML, as these tools have the potential to become integral to the effective delivery and interpretation of nanomedical technology and are tangentially related to the ethical and legal concerns surrounding nanomedical technology.

Nanotechnology has helped enhance medical treatment by improving the quality of materials using ML advancements, which, conversely, has helped realize a better understanding of nanotechnology and more effective systems of integration. These improvements have helped optimize medication efficiency, as reported by Ho et al. (2019). More recent studies, such as those conducted by Egorov et al. (2021) and Singh et al. (2020b), have shown that ML algorithms can be effectively used to anticipate the toxicity of nanomaterials. Using an example from microtechnology, microfluidic biochips developed for taking accurate blood cell counts help identify populations in cell lines, collecting information and data that may be used to more accurately identify infections due to improved integration at that scale and faster, more efficient cross-platform communication (Hassan et al., 2015; Wu et al, 2021). This indicates the links between structures at this scale and bio-detection sensitivity, which are key to the development of high-performance diagnostic instruments for disease diagnosis, and reflects how the delivery of drugs at the nanoscale may operate. Nano-biosensors have been engineered to assist in the detection of specific proteins that aid in the destruction of cancerous cells in human breast tissue (Rai et al., 2012). Nano-biosensors also help measure the efficiency of budesonide and track and compile data, which may be analyzed and tailored to personalize and improve asthma treatments. Additionally, after the magnetic clearance of nanoparticles, it is possible to perform *in situ* fluorescence technology, which can help detect SARS-CoV-2 more accurately (Cheong et al., 2020; Pramanik et al., 2020).

Given the constant evolution of nanomedical technology, it is crucial to consider the legal, social, and ethical ramifications of their broader applications as therapeutics while remaining cognizant of possible risks.

The US government launched the National Nanotechnology Initiative in 2000 to promote the responsible research and development of nanotechnology (National Nanotechnology Initiative, 2021). South Korea established National Nanotechnology Policy Center in 2010 (National Nanotechnology Policy Center, 2010), and European Union’s Horizon 2020 has proceeded nano-related programs since 2014 (European Union, 2020).

Centers for Nanotechnology in Society at the Arizona State University and the University of California, Santa Barbara, focus on research concerning the legal and ethical issues surrounding nanotechnology (Centers for Nanotechnology in Society, Arizona State University, 2021; UCSB, CNS, 2020). Despite growing interest in the ethics of nanotechnology since the early 2000s, few recent studies have comprehensively reviewed ethical and legal concerns related to the research and development of nanomedical technology. For example, Yasri and Wiwanitkit (2017) and Allon et al. (2017) conducted reviews related to recent ethical issues about nanomedicine and nanomedical technology but excluded legal aspects of issues associated with emerging nanotechnology.

Conversely, although Glenn and Boyce (2012) covered the ethical and legal dimensions of issues related to nanomedicine in their 2012 study, it is fair to assume there have been various changes in perspectives over the last decade. This review aims to highlight some of these newer issues in areas related to the research and development of nanomedical technology and identify key ethical and legal issues in the research literature related to nanomedicine.

## 2 Methods

Using scoping review methodology, this study focuses on some of the current issues being considered from various academic points of view and seeks to identify both the ethical and legal challenges highlighted by more recent breakthroughs in emergent digital nanomedicine. Scoping reviews help summarize and clarify existing research areas and identify gaps in the literature.

This review uses the methodological framework of Arksey and O’Malley (2005), using the following recommended approach: 1) identifying the research question; 2) identifying the relevant studies; 3) selecting studies for review; 4) charting data; 5) collating, summarizing, and reporting the results; and 6) consulting with experts.

### 2.1 Identifying the research question

This review aims to identify key legal and ethical issues related to nanomedicine and emerging nanomedical technology. By assessing the existing literature related to the research and development of nanomedical technology, the research question identified for this review is as follows: What are the key legal and ethical issues related to emerging nanomedical technology identified in the existing literature?

### 2.2 Identifying relevant studies

**FIGURE 1:**
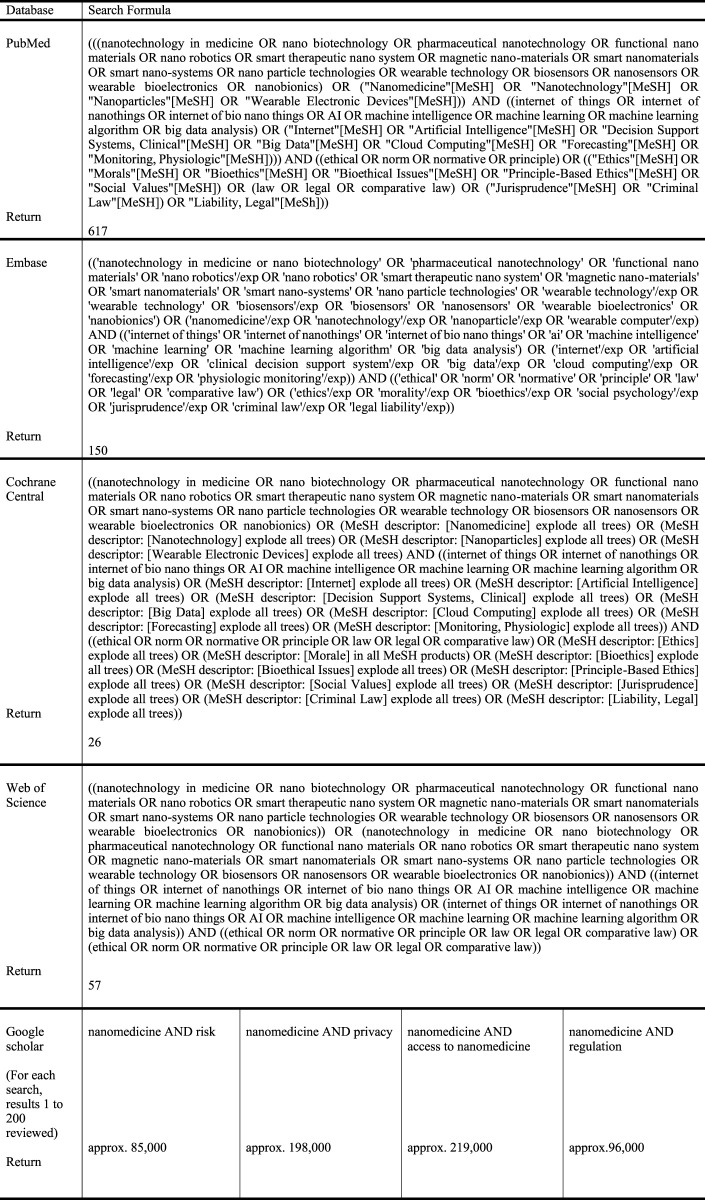
Search terms and databases used.

An initial search was performed, followed by four additional searches (Figure 1). A general search was performed on 21 October 2020 across four databases specializing in the disciplines of medicine and social sciences—PubMed, EMBASE, Cochrane Central, and Web of Science—where 617 publications were identified at PubMed, 150 at EMBASE, 26 at Cochrane Central, and 57 at Web of Science.

Articles were included for this review if they were 1) written in the English language, 2) specifically dealt with ethical or legal issues relevant to nanomedicine, and 3) were published over the last 15 years.

Articles were selected based on whether they mentioned issues related to legal and ethical aspects concerning nanomedicine or nanotechnology according to these inclusion/exclusion criteria.

This list was subsequently reviewed by all contributors to this review, and articles were re-selected based on which articles contained discussion related to legal and ethical issues specific to nanomedicine and nanotechnology. Then, this final list of articles was assessed and approved by all authors of this study.

Articles were excluded if they 1) were not written in the English language, 2) were concerned with more technical issues related to nanomedical technology or biomedical science-related issues, or 3) fell outside the 15-year time scope for inclusion in this review.

The results of these searches were limited to the literature published in English over the last 15 years, and publications concerned with biomedical or technical aspects were excluded. This produced 850 publications, where 66 duplicates were excluded and seven remained for review.

### 2.3 Selecting studies

The initial search identified four key issues: privacy, risk, access to nanomedicine, and regulation. Additional issue-oriented searches were performed on January 3, 2021, focusing on each of these individual issues, respectively, using Google Scholar to distinguish from the findings of the initial database search on PubMed, Embase, Cochrane Central, and Web of Science.

**FIGURE 2:**
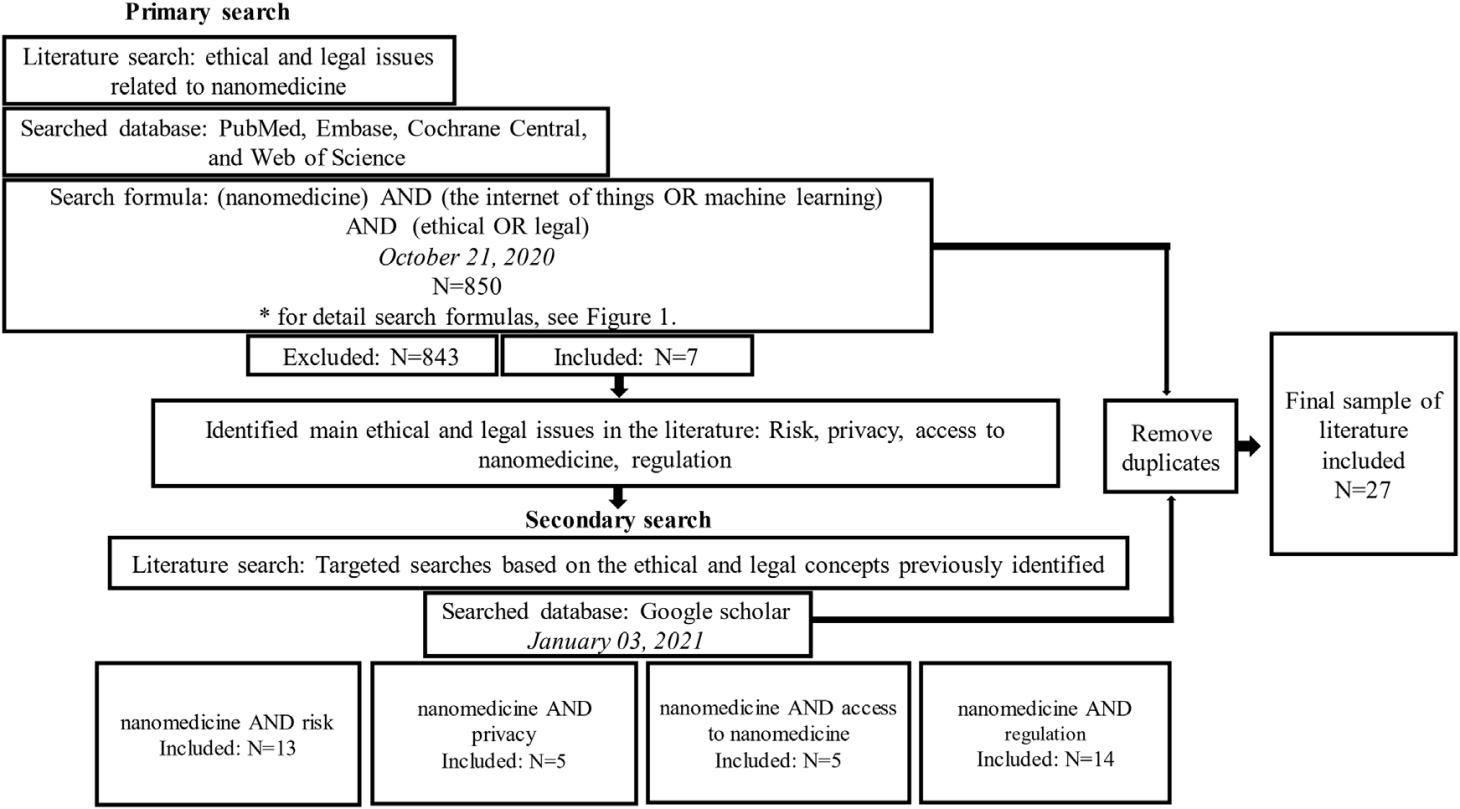
Search process flow chart.

In instances where over 200 articles were found, only the first 200 were included in the review as these were presumed most likely to be relevant to the issues. The initial search inclusion and exclusion criteria established in the previous section were applied, and an additional 17 duplicates between each independent search were removed and excluded. These searches produced 20 articles for this review, giving a total of 27 articles for analysis, which identified the following issues: exposure to harm and potential risks to health, consent issues related to nano-research and patient privacy, access to nanomedicine and nanomedical products, and classification of nanomedical devices and the precautionary principle. Figure 2 illustrates the search strategy and process followed for article selection.

### 2.4 Charting the data

**FIGURE 3:**
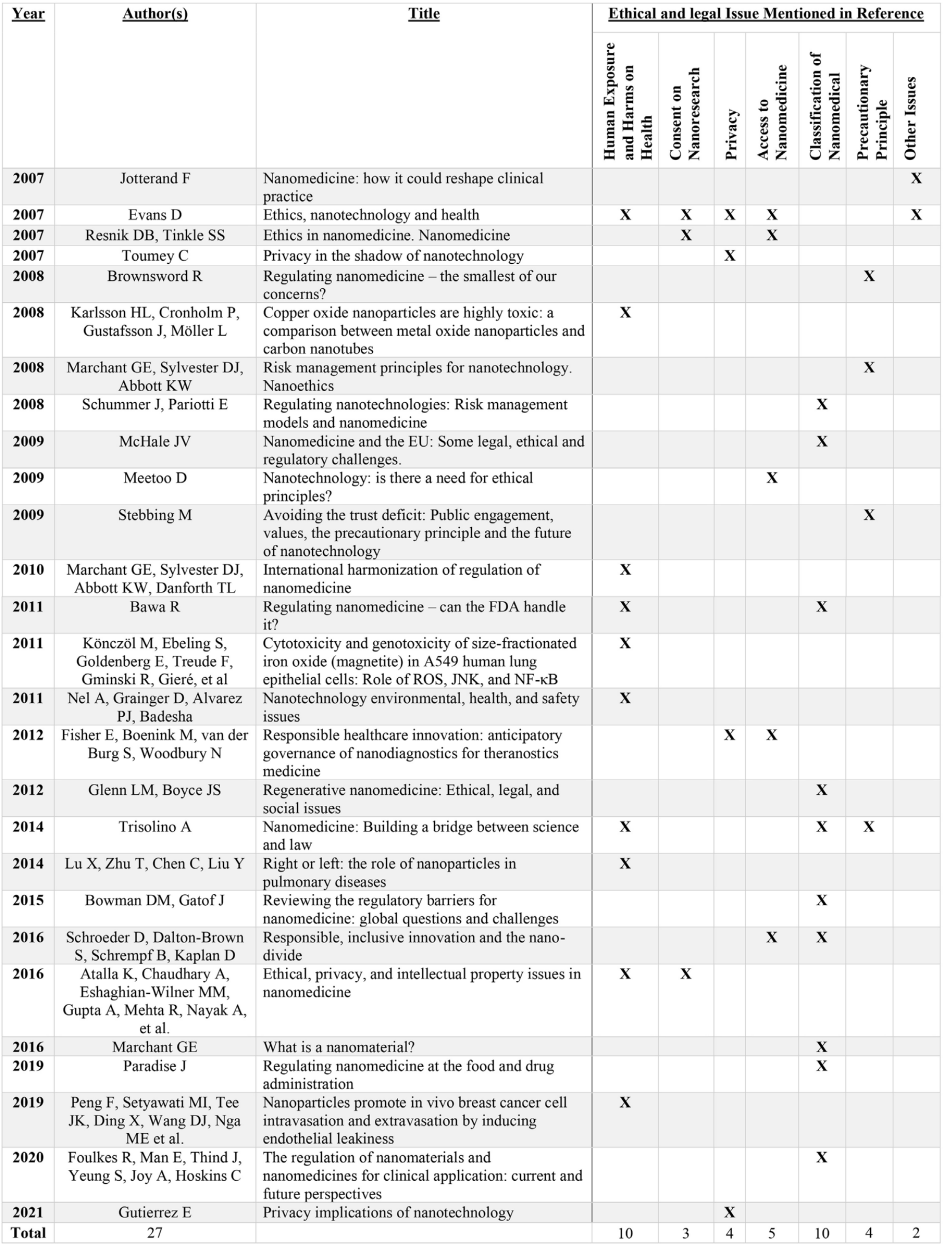
Issues identified across selected articles.

Excel was used to organize and aggregate the information most relevant to the review and then categorize it based on the nature of the ethical and legal issues raised. After the data were categorized, these key issues were examined; the differences were identified in each case, and decisions were reached as to how to best compromise on specific points of issue and resolve discrepancies. Figure 3 details the subjects discussed in the articles analyzed.

### 2.5 Collating, summarizing, and reporting results

The review results are presented in a narrative form using the methodological framework suggested by Arksey and O’Malley (2005).

Once the content relevant to this study had been identified and extracted from each article, core issues were highlighted and further analyzed from differing ethical and legal points of view, and additional recommendations were made related to the organization and presentation of results reporting these perspectives.

### 2.6 Consultation

Five experts reviewed the late-stage draft of search results, contributing their individual perspectives on methodology, medicine, biomedical technology, ethics, and law. They aided this study in determining the comprehensiveness and accuracy of the search results. Several useful recommendations were made, which helped improve the clarity and quality of this review and highlight certain limitations of the research methodology used.

## 3 Results

### 3.1 Sample characteristics

Various ethical and legal issues were identified across 27 publications spanning peer-reviewed academic journals and gray literature, where six primary areas of concern were evident: harm exposure and risks to health (*n* = 10), consent to nanomedical research (*n* = 3), privacy (*n* = 4), access to nanomedicine (*n* = 5), classification of nanomedical products (*n* = 10), and the precautionary principle (*n* = 4).

### 3.2 Harm exposure and risks to health

A key area of concern identified was the potential for harm exposure as a consequence of nanomedical technology. Nanomaterials can cause health damage due to their increased reactivity compared to their corresponding bulk form (Bawa, 2011). Clinical research has indicated that nanoparticles, such as hematite and magnetite, cause serious DNA damage (Karlsson et al., 2008; Könczöl et al., 2011), and pulmonary fibrosis can be caused by their inhalation (Lu et al., 2014). Nanomaterials may also increase the levels of toxicity in the body (Trisolino, 2014), causing blood clotting in vessels (Evans, 2007) and putting patients at an increased risk of breast cancer (Peng et al., 2019). Although it may be possible to mitigate these potential harms by discharging nanomaterials from the body, this can affect the environment instead (Atalla et al., 2016), risking potential unintended exposure and buildup, which could lead to negative consequences.

Some countries have recognized the risks posed by nanomaterials and established legal regulations related to the research and development of associated nanotechnology (Nel et al., 2011). However, international cooperation and global regulatory standards could help unify research approaches to designing, testing, and evaluating nanotechnological devices and products. Moreover, more effectively standardized guidelines help provide equal protection against harm from nanomaterials and nanotechnology across the globe (Marchant et al., 2010).

For example, Marchant et al. (2010) proposed a framework based on gradual international cooperation where mutual objectives can be agreed upon and laid out as an international convention and where plans related to nano-research and development are implemented according to agreed-upon safety protocols. If potential harm is identified, these safety protocols offer appropriate courses of action and substantive protections against the potential harm of nanomedical technology.

### 3.3 Consent issues related to nanomedical research

Research and in-depth studies are essential to the development of safe and effective nanomedical technology. However, nanomedical research presents some unique challenges for clinical researchers.

First, obtaining real informed consent effectively from participants in the proposed nanomedical device and nano-chemical drug trials may prove more difficult due to the novel and developing nature of research in the field of nanomedical devices and technology. Medical professionals may struggle to fully grasp the implications of nanomedical device-based testing because aspects are unknown or cannot be predicted regarding the behavior of nanotechnology in the human body (Resnik and Tinkle, 2007; Nel et al., 2011).

Additionally, participants in clinical research are likely to underestimate the potential risks of nano-chemical medical drugs (Resnik and Tinkle, 2007), which also presents problems in relation to obtaining fully informed consent from participants. People agreeing to engage in nanomedical research will lack sufficient context to properly consent because the technology, its associated risks, and its effects are still being studied (Atalla et al., 2016).

Particular care must be taken when communicating with research participants so that they properly understand the potential risks, although how to effectively communicate these risks to participants is unclear (Evans, 2007; Atalla et al., 2016).

### 3.4 Patient privacy

As nanomedical devices become more technologically advanced and further integrated with other types of technology, such as AI and ML, there is a concern with the increasing capacity of medical devices to generate patient data (Toumey, 2007). The detection and logging of changes in patient bodies and the collection of data to identify trends and patterns in users’ daily lives over extended periods of their lives highlight issues associated with informational asymmetry. The high sensitivity of nanomedical devices (Toumey, 2007) means nanotechnology in medicine can generate massive amounts of health data. In cases where this information may be logged to compare data groups or train AI systems, these health data could be pooled in large digital storage systems, which leaves patients in a potentially vulnerable position where their data could be used against their interests (Toumey, 2007; Fisher et al., 2012). The use of these data poses a range of potential risks and benefits for users (Toumey, 2007; Fisher et al., 2012).

In particular, the literature identifies the significance of the individual right to privacy as a potential protection against data misuse (Gutierrez, 2004), and several researchers claim that legislative actions that protect and codify the right to privacy are crucial for protecting users and patients aiding in trials contributing to the development of nanomedical technology (Gutierrez, 2004; Toumey, 2007). Since an increasingly crucial part of the right to privacy is the right to decide when and how an individual’s data will be communicated to others and used, if privacy is protected, potential harm from the misuse of information could be prevented.

Clear and specific legislative action is essential to protect patient privacy more effectively from the threat of nanotechnology.

In particular, when the primary focus of developers and decision-makers is on technological issues that can act as barriers to nanotechnology markets, legislation can bridge the gap between freedom and protection for patients and users by demanding that companies involved in the research and development of nanomedical devices retain and consult ethics advisory boards to reinforce and accommodate concepts of patient and user privacy throughout the research and development process (Gutierrez, 2004).

In addition to legislative efforts, the creative use of technology could help protect user rights to privacy, such as the decentralization of data storage.

The option of portable, inexpensive storage measures may help safeguard patient privacy, especially if these devices can store patient data locally and operate without the requirement for interfacing with massive data-sharing networks (Toumey, 2007).

### 3.5 Access to nanomedical technology

A further concern raised in the literature is unequal access. As nanomedicine is a platform for converging high-cost technology, such as nano-sensors and the so-called Internet of Things, this raises certain issues in terms of access. The high costs associated with nanomedical technology will have to be passed on to patients and will affect the cost of their healthcare coverage, especially where healthcare is governed by insurance-based health systems. These risks make nanomedical options prohibitively expensive for many patients who could see benefits from these types of technology (Resnik and Tinkle, 2007; Fisher et al., 2012).

Geographical access may also prove a challenge. Several countries, such as the Republic of Korea, Canada, and Germany, are leading innovations in nanomedicine, according to the UNESCO Science Report (Brownsword, 2008), dominating the current generation of knowledge related to nanotechnology research and development and successfully securing patents and exclusivity for these developments in nanotechnology. This emphasizes serious health service gaps between countries at the global level and raises additional concerns in relation to equity of access for patients globally, where those in the developed world will more easily be able to access nanomedical technology and those in less developed parts of the globe cannot obtain the same level of access to this type of technology. Although nanomedical technology could improve the quality of life of many patients, those living in developing countries often have far more limited access to nano-chemical drugs and nanomedical devices (Meetoo, 2009; Fisher et al., 2012).

It has become clear to researchers that unequal access to nanotechnology is an issue that must be resolved. Evans (2007), in particular, contends that humanity should share the benefits of scientific research and that global action should be taken to facilitate equality of access. Schroeder et al. (2016) also assert that when a patent regime results in one group receiving a benefit and another group experiencing a disadvantage, it is fair for the former group to share the benefit with the latter.

Reference was also made to the Responsible Research and Innovation (RRI) framework throughout the literature as a potential solution to the problem of access to nanomedical technology (Schroeder et al., 2016). RRI is a system of governance where stakeholders in new technologies work together throughout the entire research process.

Although this framework is a European initiative designed to incorporate values of inclusion, anticipation, and care for European societies, Schroeder et al. (2016) believed that RRI could be modified to include the needs of the wider global population beyond Europe. Incorporating moral values such as broader benefits for humanity could help address the problem of unequal access to nanomedical technology by making equitable access a fundamental aspect of research and development.

### 3.6 Classification of nanomedical products

As definitions of nanotechnology can be somewhat varied across the literature, the proper classification of nanotechnology presents some difficulties in relation to what types of technology can qualify as nanotechnology (Schroeder et al., 2016).

This is an issue as far as legal regulation is concerned because, without a singular definition, it becomes challenging to determine regulatory boundaries related to the research, development, and use of nanotechnology (McHale, 2009; Marchant, 2016).

The US Food and Drug Administration (FDA) has encountered additional problems related to the classification of nanotechnology (Paradise, 2019). Under the regulations of the FDA, devices undergo different examination processes to obtain premarket approval or notification. This testing depends on the potential degree of harm to health that may be caused (Paradise, 2019) by the product or the material. Nanomaterials prove difficult to classify because, due to their early-stage development, it is challenging to determine or predict potential risks (Paradise, 2019). As potential health risks are key criteria for determining the classification and the appropriate mode of examination, this causes serious regulatory problems in terms of assessing nanomedical products and technology for the FDA. Nanomaterials and their related technology also prove difficult to classify based on their primary mode of action (Schummer and Pariotti, 2008; McHale, 2009; Glenn and Boyce, 2012; Trisolino, 2014; Bowman and Gatof, 2015; Paradise, 2019; Foulkes et al., 2020). The FDA classifies products into three groups: drugs, biologics, and medical devices (Trisolino, 2014). Each classification is subject to different regulations. For instance, if the primary mode of action of a product is chemical, the FDA applies the classification requirements of drug criteria to the product. Researchers have noted that the FDA’s criteria for product classification cannot be applied to nanomedicine because, at the atomic and molecular levels, whether the primary mode of action of a product is chemical, mechanical, or electrical is not distinguishable (Schummer and Pariotti, 2008; McHale, 2009; Bawa, 2011; Glenn and Boyce, 2012; Bowman and Gatof, 2015; Paradise, 2019; Foulkes et al., 2020).

To address this problem, Bowman and Gatof (2015) proposed that regulators improve their expertise and understanding of nanotechnology and that active communication occur between regulators and producers during the product approval process (Bowman and Gatof, 2015). Additionally, as current classifications lack the specificity to properly categorize nanotechnology, they should be re-assessed and revised to allow for their proper classification. Trisolino (2014) suggested that if nanomedical products are classified depending on whether their primary functions are repair, replacement, or augmentation, this may remedy the current regulatory challenges faced by the FDA (Trisolino, 2014).

### 3.7 The precautionary principle

The precautionary principle refers to the widely accepted approach to the regulation of innovation and new technology in which the potential for harm has not yet been properly investigated or is otherwise not yet known (Stebbing, 2009).

In terms of nanomedical technology, this means that decision-makers should delay the use of new nanomedical technology until it can be ensured that these new types of technology are not causing risks to public health or the environment beyond an acceptable level (Brownsword, 2008; Marchant et al., 2008).

This review identified two critical concerns in relation to regulating nanomedical technology according to the precautionary principle. First, the principle is ultimately limited in guiding decision-making due to a lack of clarity and vaguely defined standards (Marchant et al., 2008). It is possible, for instance, to use the precautionary principle to support either side of an issue, depending on how hazard or risk is defined in any given case (Trisolino, 2014), and an accurate estimation of the level of harm that a specific technology could cause is difficult (Brownsword, 2008). Second, the precautionary principle is biased toward preserving the *status quo*, so it may act as an impediment to the development and deployment of new technology (Marchant et al., 2008).

Marchant et al. (2008) proposed an alternative to the precautionary principle based on a model introduced by Ayres and Braithwaite (1992). According to this alternative model, regulatory criteria are applied to nanomedical technology based on the level of risk knowledge (Brownsword, 2008). For instance, at the early stages of risk assessment, nanotechnology developers exercise self-imposed standards of risk management using extensive cautionary measures and reasonable degrees of self-regulation. As knowledge and understanding of potential risks accumulate, the industry can establish standards for the regulation of the safe research and development of nanomedical technology.

Another proposed alternative to the precautionary principle is interactive participatory approaches (Stebbing, 2009) to determining risk. These approaches involve key stakeholders, such as scientists, lawmakers, policymakers, and members of the wider public, working together to determine the degrees of risk posed by new technology, incorporating various dimensions of socioeconomic concerns caused by these new types of technology, and designing the most effective management solutions.

### 3.8 Other issues

Issues pertaining to doctor-patient relationships, data ownership, and diagnosis without treatment related to nanomedical technology were mentioned at points throughout the compiled literature, although not frequently enough to draw any general conclusions in relation to nanomedicine and nanomedical technology. In terms of doctor-patient relationships, nanomedical technology proposes a possible issue for doctors, as nano-sensors may act as a personal source of bio-information for patients, allowing them some degree of agency when assessing and monitoring their health. In cases where information provided by sensors may conflict with information provided by medical professionals, patients may suffer distress or frustration; consequently, their faith and trust in their doctors are compromised (Jotterand, 2007).

Similarly, Bert noted that nanomedical technology can detect diseases that may not yet be curable, which risks causing psychological harm to patients when care and caution are not properly exercised (Evans, 2007). A final issue addressed briefly in the literature is personal ownership of bio-information and biodata. When nano-sensors are used to gather and analyze genetic data, a determination of not only the individual’s genetic characteristics but also the genetic characteristics of that individual’s relatives may be possible.

This poses questions as to what extent a person has ownership and rights over their familial genetic data and the degree to which they exercise any control over how these data are used and what happens to them (Evans, 2007).

## 4 Discussion

This review attempts to demonstrate various legal and ethical issues surrounding nanomedicine and nanomedical technology. Although there is a general degree of consensus on specific issues, the main problem appears to be a lack of specificity as to what form solutions to these issues will take. For example, studies were explicitly clear about the importance of care and clarity in obtaining proper informed consent from participants in nanomedical research. However, they were far less clear about how to properly inform and obtain consent from participants.

Similarly, although the introduction of a framework convention was suggested as the most effective form of governance and global cooperation, there were no specifications for any structure or mandatory requirements for participation, which limits the practical use of any framework convention to address the issues of potential harm to health.

Concerning issues related to privacy protection, the decentralization of information-gathering systems was suggested as a potential solution to the problem. This mechanism, in particular, may help protect privacy because decentralized health data circuits make it harder for unauthorized parties to access data. Conversely, data centralization aids in the extraction of valuable information from data, so the decentralization of information-gathering systems necessarily limits abilities to retrieve valuable information. Consequently, proponents of decentralization must specify at which points it is possible to achieve a level of optimal balance with data centralization. When limits are clearly defined, medical professionals can protect patient privacy while gathering valuable information about specific conditions and their symptoms.

This review also highlighted that the majority of the English-language discussion surrounding the classification of nanomedical technology is focused primarily on the United States and uses the regulatory systems of the FDA as an example to illustrate the problems associated with classifications of nanomedical technology. However, several nations, including the Republic of Korea, Germany, and Canada, are also world leaders in the research and development of nanomedical technology (UNESCO Science Report, 2015). As the use and application of new technology can cause potential legal issues in any jurisdiction, it may prove useful to compare the regulatory approaches of these nations, which could aid in working toward more comprehensive, useful means of classifying nanomedical products.

Searches did not generate any articles that dealt specifically with ethical and legal issues related to nanomedical technology and its relationship to ML. However, evidence suggests that ML and its related literature can offer answers to several nanomedicine-related problems.

Research indicates that ML performs exceptionally well in predicting the toxicity of nanomaterials (Jotterand, 2007; Marchant et al., 2008; UNESCO Science Report, 2015; Winkler, 2020) and that medical professionals could assess the toxicity of nanoparticles by applying ML in the realm of nanomedical technology (Luechtefeld et al., 2018). Additionally, as literature concerning ML has conducted in-depth investigations on data ownership, researchers can consult this literature to discover answers to ownership-related problems (Singh et al., 2020a). Although ML may be useful when considering some nanomedicine-related issues, researchers should exercise some level of caution as ML algorithms can complicate issues in nanomedicine.

For instance, a large amount of data must be obtained and stored to train ML algorithms. Due to the centralized nature of storage and processing, data providers may face the threat of identification, compromise, or other types of exploitation (Price, 2017; Jaremko et al., 2019; Racine et al., 2019; Brady and Neri, 2020; Safdar et al., 2020).

Researchers also noted the potential threat to aspects of trust in the relationship between doctors and patients, as patients might start to trust their nanomedical devices more than their doctors. However, in this case, there was no discussion of any potential resolution to this issue, and given that the relationships of trust are vital to medical practice, further research is necessary to explore this potential problem and the effects of technology and personal agency on the doctor-patient relationship, especially as personal health monitoring devices continue to become more accessible to patients.

### 4.1 Limitations

PubMed, EMBASE, Cochrane Central, and Web of Science were not used for additional searches as these search engines were already used for the initial, general searches. Instead, Google Scholar was used for further issue-focused searches.

This strategy may prove a significant limitation of this review for several reasons. For example, Google Scholar may have missed articles that the other four search engines would have discovered. A second issue is that Google Scholar has more limitations than other databases in terms of search reproducibility. Finally, only the first 200 items were examined, although more than 200 articles were related to certain subjects.

Additionally, due to the time scale involved in identifying, compiling, and collating the data relevant to this review, it is entirely possible that there are more recent developments in the discourse relating to the ethical, legal, and social implications of nanomedical innovations in the years following its publication, outside the scope of this study. A further limitation of this study is that by limiting selections to English-language articles, there is the possibility that additional information and alternative perspectives covered in other languages have not been considered in this review. Because of these specific issues, the conclusions drawn by this review may not be entirely optimal. However, they may help provide useful insights into future research concerning areas related to nanomedical technology.

## 5 Conclusion

This review attempts to effectively demonstrate that nanomedicine has received considerable attention across academic literature and highlights the various solutions proposed to address legal and ethical concerns related to the research, development, and use of nanomedical technology.

First, it is evident that several ethical, legal, and social issues associated with nanomedical technology are closely linked with other forms of technology. Consequently, many key concerns related to nanomedical technology are tangentially associated with the potential ethical implications of AI and ML and the potential impact of the “Internet of Things” on healthcare systems and medicine-related issues. There are opportunities to interrogate the nature of this relationship in more detail and assess the potential risks and benefits associated with basing health monitoring and treatments on hybrid technologies that are interdependent to function. Although concerns surround the ability to consent to nanomedical research, a small amount of the literature reviewed was focused on consent to data ownership and distribution.

This is a potential problem because a patient may choose to consent to their involvement in nanomedical research but not consent to the use of the biodata generated being used in training AI models or aiding in ML, so further investigation is required regarding the level of consent required from patients with a view to protecting their data interests and understanding the relationship between these different technologies.

There are also significant opportunities for research into the nature of these links with nanomedical technology, their effects, and how their integration with healthcare and medicine may affect the nature of ethical caregiving.

For example, although the potential impacts on the doctor-patient relationship were briefly called into question by researchers in this review, further research could be undertaken to assess public attitudes in relation to the use of nanomedical technology, as there is a possibility that patients refrain from engaging with nanomedical technology due to wariness and fear of the potential effects.

In terms of classification, some of the ideas presented in this review may be useful as a starting point for developing more appropriate and effective legal frameworks governing the legal and ethical environment surrounding nanomedical technology. However, some of the proposed solutions to these issues lack sufficient specificity for any practical effect.

The fact that there are no clear legal guidelines on the use and level of acceptable risk that may be tolerated in a healthcare setting compromises the possibility of innovation in potential treatment areas due to vague and unspecific criteria currently used for their assessment and places patients at risk of unnecessary harm as a result. Future trials of nanomedical products and technology should focus on understanding the nature and measurability of these potential harms so that law and policymakers and, by extension, product developers and manufacturers can set appropriate and specific safety standards for the research, development, and use of nanomedical technology that can be properly classified and tested. Furthermore, regulatory issues in this review are primarily concerned with the legal frameworks governing nanomedical technology in individual countries. Therefore, special attention is required when assessing the validity of these suggestions at the global level to prevent the reinforcement of structural disparities, and it is crucial to standardize practices and ensure equal access to nanomedical technology on a worldwide scale.

## Data Availability

The original contributions presented in the study are included in the article/Supplementary Material; further inquiries can be directed to the corresponding author.
