# Supplementary material for: Ethical and legal challenges in nanomedical innovations: a scoping review
Source: Front Genet. 2023 May 12;14:1163392. doi: 10.3389/fgene.2023.1163392 (PMC10213273; doi:10.3389/fgene.2023.1163392)
Supplement: Supplementary file 1 [file DataSheet1.PDF]

Additional File 3: List of coded sources and issues, by source

**Year**	**Author(s)**	**Title**	**Ethical and legal Issue Mentioned in Reference**						
			**Harm Exposure and Risks to Health**	**Consent to Nanomedical Research**	**Privacy**	**Access to Nanomedicine**	**Classification of Nanomedical Products**	**Precautionary Principle**	**Other Issues**
**2007**	Jotterand F	Nanomedicine: how it could reshape clinical practice							**X**
**2007**	Evans D	Ethics, nanotechnology and health	**X**	**X**	**X**	**X**			**X**
**2007**	Resnik DB, Tinkle SS	Ethics in nanomedicine. Nanomedicine		**X**		**X**			
**2007**	Toumey C	Privacy in the shadow of nanotechnology			**X**				
**2008**	Brownsword R	Regulating nanomedicine – the smallest of our concerns?						**X**	
**2008**	Karlsson HL, Cronholm P, Gustafsson J, Möller L	Copper oxide nanoparticles are highly toxic: a comparison between metal oxide nanoparticles and carbon nanotubes	**X**						
**2008**	Marchant GE, Sylvester DJ, Abbott KW	Risk management principles for nanotechnology. Nanoethics						**X**	
**2008**	Schummer J, Pariotti E	Regulating nanotechnologies: Risk management models and nanomedicine					**X**		
**2009**	McHale JV	Nanomedicine and the EU: Some legal, ethical					**X**		

Additional File 3: List of coded sources and issues, by source

Year	Author(s)	Title	Ethical and legal Issue Mentioned in Reference						
			Harm Exposure and Risks to Health	Consent to Nanomedical Research	Privacy	Access to Nanomedicine	Classification of Nanomedical Products	Precautionary Principle	Other Issues
		and regulatory challenges							
**2009**	Meetoo D	Nanotechnology: is there a need for ethical principles?				**X**			
**2009**	Stebbing M	Avoiding the trust deficit: Public engagement, values, the precautionary principle and the future of nanotechnology						**X**	
**2010**	Marchant GE, Sylvester DJ, Abbott KW, Danforth TL	International harmonization of regulation of nanomedicine	**X**						
**2011**	Bawa R	Regulating nanomedicine – can the FDA handle it?	**X**				**X**		
**2011**	Könczöl M, Ebeling S, Goldenberg E, Treude F, Gminski R, Gieré, et al	Cytotoxicity and genotoxicity of size-fractionated iron oxide (magnetite) in A549 human lung epithelial cells: Role of ROS, JNK, and NF-κB	**X**						
**2011**	Nel A, Grainger D, Alvarez PJ, Badesha	Nanotechnology environmental, health, and safety issues	**X**						

Additional File 3: List of coded sources and issues, by source

Year	Author(s)	Title	Ethical and legal Issue Mentioned in Reference						
			Harm Exposure and Risks to Health	Consent to Nanomedical Research	Privacy	Access to Nanomedicine	Classification of Nanomedical Products	Precautionary Principle	Other Issues
**2012**	Fisher E, Boenink M, van der Burg S, Woodbury N	Responsible healthcare innovation: anticipatory governance of nanodiagnostics for theranostics medicine			**X**	**X**			
**2012**	Glenn LM, Boyce JS	Regenerative nanomedicine: Ethical, legal, and social issues					**X**		
**2014**	Trisolino A	Nanomedicine: Building a bridge between science and law	**X**				**X**	**X**	
**2014**	Lu X, Zhu T, Chen C, Liu Y	Right or left: the role of nanoparticles in pulmonary diseases	**X**						
**2015**	Bowman DM, Gatof J	Reviewing the regulatory barriers for nanomedicine: global questions and challenges					**X**		
**2016**	Schroeder D, Dalton-Brown S, Schrempf B, Kaplan D	Responsible, inclusive innovation and the nano-divide				**X**	**X**		
**2016**	Atalla K, Chaudhary A, Eshaghian-Wilner MM, Gupta A, Mehta R, Nayak A, et al.	Ethical, privacy, and intellectual property issues in nanomedicine	**X**	**X**					
**2016**	Marchant GE	What is a nanomaterial?					**X**		

Additional File 3: List of coded sources and issues, by source

Year	Author(s)	Title	Ethical and legal Issue Mentioned in Reference						
			Harm Exposure and Risks to Health	Consent to Nanomedical Research	Privacy	Access to Nanomedicine	Classification of Nanomedical Products	Precautionary Principle	Other Issues
**2019**	Paradise J	Regulating nanomedicine at the food and drug administration					**X**		
**2019**	Peng F, Setyawati MI, Tee JK, Ding X, Wang DJ, Nga ME et al.	Nanoparticles promote in vivo breast cancer cell intravasation and extravasation by inducing endothelial leakiness	**X**						
**2020**	Foulkes R, Man E, Thind J, Yeung S, Joy A, Hoskins C	The regulation of nanomaterials and nanomedicines for clinical application: current and future perspectives					**X**		
**2021**	Gutierrez E	Privacy implications of nanotechnology			**X**				
**Total**	27		10	3	4	5	10	4	2
